# Subchronic Low-Dose Methylmercury Exposure Accelerated Cerebral Telomere Shortening in Relevant with Declined Urinary aMT6s Level in Rats

**DOI:** 10.3390/toxics11020191

**Published:** 2023-02-18

**Authors:** Xi Wu, Ping Li, Junyan Tao, Xiong Chen, Aihua Zhang

**Affiliations:** 1 The Key Laboratory of Environmental Pollution Monitoring and Disease Control, Ministry of Education, School of Public Health, Guizhou Medical University, Guiyang 550025, China; 2 State Key Laboratory of Environmental Geochemistry, Institute of Geochemistry, Chinese Academy of Sciences, Guiyang 550081, China

**Keywords:** methylmercury, telomere shortening, melatonin, 6-sulfatoxymelatonin, 5-hydroxytryptamine

## Abstract

Methylmercury (MeHg) is a global pollutant with established toxic effects on the central nervous system (CNS). However, early events and early-warning biomarkers of CNS damage following exposure to low-dose MeHg are still lacking. This study aimed to investigate whether subchronic low-dose MeHg exposure had adverse effects on the cerebral telomere length, as well as serum melatonin and its urinary metabolite 6-sulfatoxymelatonin (aMT6s) in rats. Sixteen male Sprague Dawley rats were divided into two groups. Group I was the control group. In group II, rats were exposed to MeHg by gavage at a dose of 0.1 mg/kg/day for 3 months. This study revealed that MeHg exposure resulted in impairment of learning and memory ability, a slightly reduced number of neurons and an irregular arrangement of neurons in the hippocampus. It also significantly accelerated telomere shortening in the cerebral cortex, hippocampus and hypothalamus. Moreover, MeHg exposure decreased the levels of melatonin in serum and aMT6s in urine, partly by suppressing the synthesis of 5-hydroxytryptamine (5-HT) in the brain but promoted the expression of melatonin-catalyzing AANAT and ASMT. Importantly, cerebral telomere length was positively correlated with MT and aMT6s after MeHg exposure. These results suggested that the shortened telomere length in the brain may be an early event in MeHg-induced CNS toxicity, and the level of aMT6s in urine may serve as an early-warning biomarker for MeHg-induced CNS damage.

## 1. Introduction

Mercury is known to be a widespread environmental contaminant and one of the most toxic heavy metals detectable in the environment [1]. Even at low concentrations, mercury exposure can cause a variety of health problems [2,3]. It has been widely used in metallurgy, scientific measuring instruments (such as thermometers and barometers), dental amalgam fillers and other manufacturing activities, and can enter the human body in various ways, such as the atmosphere, soil, water and food [4,5], seriously threatening human health. Methylmercury (MeHg) is one of the most toxic forms of mercury, with particularly adverse effects on the central nervous system (CNS). Subchronic MeHg exposure has been reported to affect brain development and cause motor and cognitive impairments in children, as well as neurological damage and neurodegenerative disease in adults [6,7,8]. Nervous system damage caused by MeHg is related to the fact that the brain is a highly oxygen-consuming organ and, therefore, more prone to oxidative stress [9]. Moreover, MeHg can also reduce the antioxidant activity of cells by interacting directly with antioxidants or selenium [10].

Telomeres are sequences of genes found at the ends of chromosomes and are responsible for maintaining genome integrity [11]. A shorter telomere length predicts an increasing risk of disease [12]. An increasing body of evidence suggests that the shortened telomere length may reflect the adverse health effects of environmental pollutants [13,14]. Studies have shown that the generation of reactive oxygen species and consequent oxidative stress is an important toxic mechanism of MeHg [4]. Excessive oxidative stress can induce telomere damage, especially to the repetitive series structure, which is rich in base G and causes telomeres to break and shorten under non-replication conditions [15]. However, whether MeHg has a negative effect on cerebral telomere length is unclear.

Melatonin is a neuroendocrine hormone that is primarily synthesized in the pineal gland and mainly synthesized by 5-hydroxytryptamine (5-HT) under the continuous catalysis of arylalkylamine-N-acetyltransferase (AANAT) and acetylserotonin O-methyltransferase (ASMT) [16,17]. It is a powerful endogenous antioxidant that is effective in preventing oxidative-stress-induced cellular oxidative damage [18]. Importantly, melatonin, when synthesized under physiological conditions with a circadian rhythm, has been shown to have a protective effect regarding mercury-induced CNS damage [19,20,21]. Therefore, it is significant to investigate the potential impact of MeHg on endogenous melatonin secretion for the prevention and treatment of MeHg-induced CNS damage.

Melatonin synthesis and secretion are reflected by detecting melatonin levels in peripheral blood; this collection is difficult and invasive. In vivo, melatonin is catalyzed into 6-hydroxymelatonin by liver microsomal hydroxylase, and nearly 60–80% of 6-hydroxymelatonin binds to sulfate to form 6-sulfatoxymelatonin (aMT6s) [22]. At present, there is a growing body of evidence suggesting that urinary aMT6s levels could reflect the circulating melatonin level [23,24]. Thus, melatonin secretion can also be evaluated by detecting the urinary aMT6s level.

Therefore, this study aimed to investigate the adverse effect of subchronic low-dose MeHg exposure on CNS and its underlying mechanism in rats, including cerebral telomere length and melatonin secretion level, and to analyze the association between urinary aMT6s level and cerebral telomere length. This study provides evidence of urinary aMT6s level as an effective early-warning biomarker to assess MeHg-induced CNS damage, as well as providing insight regarding the prevention of CNS damage in populations exposed to MeHg.

## 2. Materials and Methods

**Warning:** The use of methyl mercury is extremely hazardous and requires special precautions during handling to reduce the risk of harm.

### 2.1. Animals and Experimental Design

Male Sprague Dawley rats weighing 180–200 g were purchased from and maintained in the Experimental Animal Center of Guizhou Medical University (Guiyang, China) The rats were provided with commercial feed obtained from Henan Huanyu Hekang Biotechnology Co., Ltd (Anyang, China). The feed contained corn, fish meal, flour, vegetable oil, vitamins and trace elements, amino acids, etc. During the feeding process, the rats drank and ate ad libitum under a 12-h light/12-h dark cycle; the temperature of the rats’ housing environment was 22 ± 2 °C. All animals were acclimatized to the facility for 7 days before the experiment began. Sixteen rats were divided into two groups, with eight rats in each group: a control group and MeHg exposure group. The MeHg (CH_3_HgCl, Sigma-Aldrich, Helsinki, Finland) was administered by gavage at a dose of 0.1 mg/kg/day (equivalent to 0.116 mg/kg/day of CH_3_HgCl). The dose of MeHg (0.1 mg/kg/day) was selected based on a previous study of the daily MeHg ingestion by Brazilian Riparian communities exposed to MeHg through contaminated fish intake [25]. Although the dose is not exactly comparable to that in fish-eating communities, it is much more representative of environmental MeHg exposure than traditional studies of the effects of MeHg exposure in animals. Exposure lasted 3 months, which is representative of subchronic exposure in rats.

During the experiments, the weights of rats were recorded weekly. Rat urine was collected at 19:00–22:00/22:00–1:00/1:00–4:00/4:00–7:00 time periods for aMT6s analysis one week before the rats were sacrificed. After feeding for 3 months, rats were anesthetized with 3% pentobarbital sodium, and blood samples were taken from the heart. Half of each rat’s blood was collected and placed in the anticoagulant tube to detect Blood-Hg concentrations; the rest was placed in a non-anticoagulant tube, which was stored at room temperature for 1 h and centrifuged for 20 min at 1000× *g* at 4 °C; then, the supernatant was removed to test the serum melatonin level. Pineal glands were homogenized to detect the mRNA level of AANAT and ASMT. Rat brains were weighed, washed and dissected into two portions. The brain somatic index was calculated using the formula (100 × [brain weight (g)/body weight (g)]). Half of the brain was fixed in 4% paraformaldehyde solution for later histological analysis. The rest of the brain tissue was separated into three portions (cortex, hippocampus and hypothalamus), half of each portion was used to detect telomeres, and the other half was used to detect the level of 5-HT.

All experiments and procedures associated with this study were performed in accordance with guidelines for animal care and use and approved by the ethics committee of the Guizhou Medical University (Ethics No. 2200493).

### 2.2. Morris Water Maze

Behavioral testing was performed using the Morris water maze. The assay was performed based on the methods reported in a previous study [26]. The black circular pool (120 cm in diameter and 60 cm in height) was filled with tap water (22 ± 1 °C) until the escape platform (10 cm diameter) was 2 cm below the surface.

Positioning and navigation experiment: First, the pool was divided into four quadrants; the platform was located in the fourth quadrant, 2 cm below the water level. The time provided to the rat to find the hidden platform was 120 s, and if it failed, it was gently guided to the platform and was allowed to remain there for 20 s. The training lasted for 4 days. Space search experiment: On the 5th day, the platform was removed, one quadrant was chosen, and the rat surface wall went into the water. In the absence of the platform, the time taken and the first time reached the platform of the rats were recorded.

### 2.3. ELISA

After adding RIPA lysis buffer, the cortex, hippocampus and hypothalamus tissue were, respectively, placed into the grinder to be grinded. The supernatant was taken, and total protein concentration was determined with the BCA kit (Solarbio, Beijing, China). The sample was diluted with PBS to ensure that the total protein in each test hole of 5-HT did not exceed 0.3 mg. The urine collected at each time was centrifuged at 4000 rpm for 10 min, and the supernatant was then taken. MT levels (Elabscience, Wuhan, China) in serum, aMT6s levels (RunYu, Shanghai, China) in urine and 5-HT levels (FineTest, Wuhan, China) in brain were measured using commercially available enzyme-linked immunosorbent assay (ELISA) kits according to the manufacturer’s instructions.

### 2.4. Hematoxylin–Eosin (HE) Staining

After fixation with 4% paraformaldehyde for 24 h, the brain tissue was embedded in paraffin and sliced to 4 µm. Paraffin sections were dewaxed twice in xylene, dyed with hematoxylin solution and washed with distilled water to remove floating color. The differentiation solution was rinsed twice with tap water after differentiation. Then, each section was dyed with eosin solution, dehydrated with anhydrous ethanol, made transparent with xylene, and sealed with a neutral gum. The images were obtained using a microscope (Nikon Eclipse E100, Tokyo, Japan).

### 2.5. Nissl Staining

The fixed brain tissue was washed with running water and immersed in paraffin for embedding. Slice thickness was 5 µm, and slicers were dewaxed to water. The sections were placed in Cresyl violet stain, and the dye tank was immersed in a 56 °C box for 1 h. Each section was placed in Nissl Differentiation for a few seconds (until the background was nearly colorless). Each section was dehydrated, made transparent and sealed. Image acquisition was finished using a microscope (Nikon Eclipse E100, Tokyo, Japan).

### 2.6. Determine Blood-Hg Concentrations

Whole-blood samples were taken and placed in a 25 mL borosilicate glass colorimetric tube. Then, 5 mL of HNO_3_ was added, acid-treated glass spheres were placed in the colorimetric tube, and samples were digested in water bath at 95 °C for 3 h. After cooling, a small amount of ultra-pure water was added first, and then 0.5 mL of BrCl was added. About 24 h later, 2–3 drops of NH_2_OH·HCl solution were added to create yellow recede, and ultra-pure water was added at a constant volume to 25 mL. The method was reduced by SnCl_2_ and determined by cold atomic fluorescence spectrometry (AF-630A, Beijing, China).

### 2.7. Real-Time Quantitative PCR

Total RNA was extracted from the pineal tissue using TRIzol reagent (Invitrogen, Carlsbad, CA, USA), according to the manufacturer’s instructions. Complementary DNA was synthesized using a RevertAid First Strand cDNA Synthesis Kit (Thermo Fisher Scientifific, Vilnius, Lithuania). qPCR for AANAT, ASMT and GAPDH was performed using a Real-Time PCR Detection System. Primer sequences used for qPCR were as follows: forward AANAT 5′-GTG GCT GCT GAC CCA AG-3′, reverse AANAT 5′-TGC TGT CTC CCT TCA TGC T-3′, forward ASMT 5′-GTG CCT GCG TGG AGT TG-3′, reverse ASMT 5′-CCA TGA CCC TGT GAC CCT-3′, forward GAPDH 5′-TCT CTG CTC CTC CCT GTT C-3′, and reverse GAPDH 5′-ACA CCG ACC TTC ACC ATC T-3′. PCR amplifications were performed in a total volume of 20 µL using SuperReal PreMix (SYBR Green) (Tiangen) with an iCycler thermal cycler (Bio-Rad, CFX96^TM^ Optics Module). The gene expression level was calculated using the 2^−∆∆Ct^ method, and the relative AANAT and ASMT level was normalized to that of GAPDH.

### 2.8. Genomic DNA Isolation and Telomere Length Analysis

Genomic DNA was extracted from the PBMCs isolated from whole blood using TIANamp Genomic DNA Kit (Tiangen, Beijing, China). DNA concentration was measured using the microplate reader. Samples were diluted to a final concentration of 25 ng/1.5 µL to measure telomere length. qPCR was performed using SuperReal PreMix (SYBR Green) (Tiangen, Beijing, China). Primers used were as follows: forward TEL 5′-GGT TTT TGA GGG TGA GGG TGA GGG TGA GGG TGA GGG t-3′, reverse TEL 5′-TCC CGA CTA TCC CTA TCC CTA TCC CTA TCC CTA TCC CTA-3′, forward AT1 5′-ACG TGT TCT CAG CAT CGA CCG CTA CC-3′, and reverse AT1 5′-AGA ATG ATA AGG AAA GGG AAC AAG AAG CCC-3′. The relative telomere length was measured by comparing the ratio of telomere repeat copy number (T as Tel1) and single gene copy number (S as AT1), expressed as telomere length (T/S) ratio. Each individual value obtained by qPCR was processed through the formula T/S = 2^−∆Ct^, where ΔCT = CT_telomere_ − CT_AT1_. This ratio was then compared with the ratio of the reference DNA. Each DNA sample collected was measured in duplicate.

### 2.9. Statistical Analysis

For parametric data, an unpaired Student’s t-test was used when there were two groups. For nonparametric data, a Mann–Whitney test was performed when there were two groups. The Spearman correlation coefficient test was used to analyze the correlation between all parameters. The data were expressed as mean ± standard deviation (SD). Differences between groups were considered significant at *p* < 0.05. Statistical analyses were performed using GraphPad Prism software (version 6.0; GraphPad Software Inc., San Diego, CA, USA).

## 3. Results

### 3.1. Influence of MeHg on Blood Mercury Concentration and Brain Somatic Index in Rats

**Figure 1:**
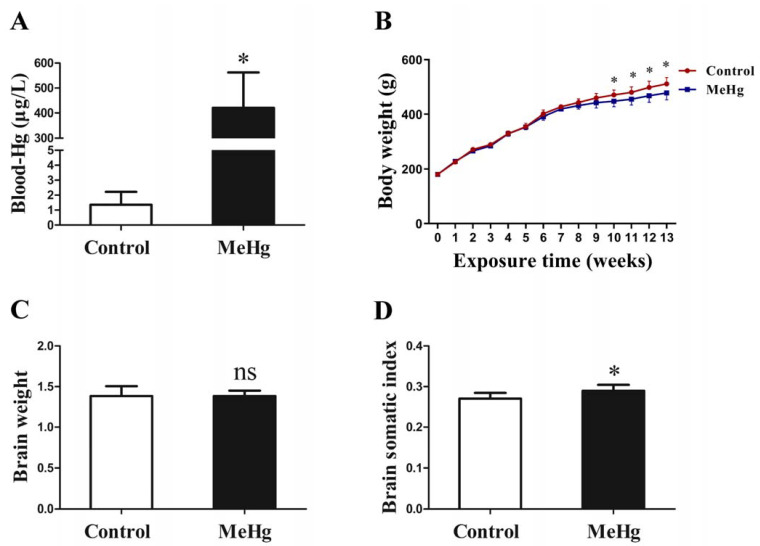
Impact of 0.1 mg/kg/day MeHg exposure for 3 months on mercury concentrations in blood (**A**), body weight (**B**), brain weight (**C**) and brain somatic index (**D**) in rats. Blood-Hg, blood mercury concentration; MeHg, methylmercury. The data are expressed as mean ± SD for eight rats per group. ns, no significance compared to the control group, *p* > 0.05. * *p* < 0.05, significant difference compared to the control group.

To determine the internal load of MeHg in rats, the blood mercury concentration was detected. Compared with the control group, MeHg exposure for 3 months induced a significant increase in the mercury concentration in rat blood (*p* < 0.05; Figure 1A). To assess the effect of MeHg on body growth and brain development, the body weight and brain weight were measured. The body weight of MeHg-exposed rats was significantly lower than that of the control group from the 10th week (*p* < 0.05; Figure 1B). MeHg exposure did not show an obvious effect on brain weight (*p* > 0.05; Figure 1C) but increased brain somatic index (*p* < 0.05; Figure 1D).

### 3.2. The Effect of MeHg on Spatial Learning and Memory in Rats

**Figure 2:**
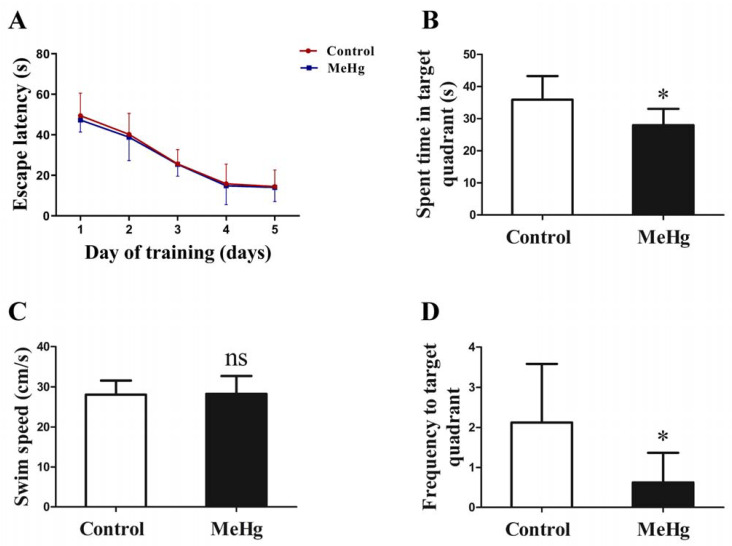
The influence of MeHg exposure on spatial learning and memory in the Morris water maze, including the escape latencies of the groups during the training trials (**A**), time spent in target quadrant (**B**), swimming speed (**C**), and the frequency of entering the target quadrant (**D**). The data are expressed as mean ± SD for eight rats per group. ns, no significance compared to the control group, *p* > 0.05. * *p* < 0.05, significant difference compared to the control group.

To explore the impacts of MeHg on spatial learning and memory, Morris water maze tests were performed. There was no statistically significant difference between the escape latency result of the two groups (*p* > 0.05; Figure 2A). The probe test was performed on the last day of MeHg exposure. The results showed that MeHg-treated rats spent less time in the target quadrant than the control group (*p* < 0.05; Figure 2B), but the difference in swimming speed between the two groups was not statistically significant (*p* > 0.05; Figure 2C). However, MeHg significantly decreased the frequency of entering the target quadrant (*p* < 0.05; Figure 2D). These results suggest that subchronic low-dose MeHg exposure could impair spatial memory performance in rats.

### 3.3. Impact of MeHg on Pathological Morphology and Telomere Length of Rat Brain Tissue 

**Figure 3:**
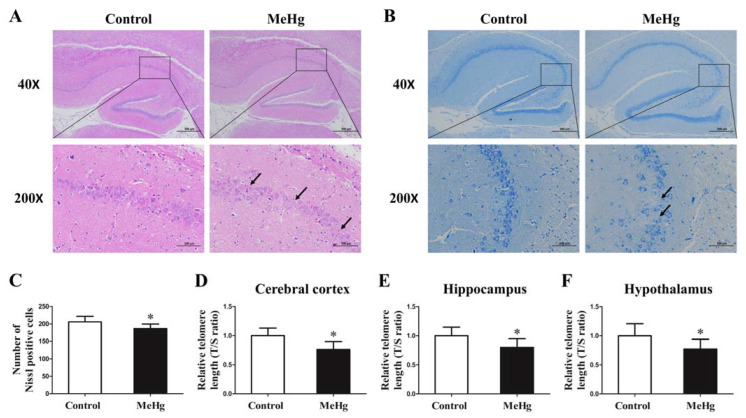
The influence of MeHg exposure on pathological morphology of rat brain tissue detected by HE staining (**A**) and Nissl staining (**B**,**C**) as well as the telomere length in cerebral cortex (**D**), hippocampus (**E**) and hypothalamus (**F**). The pyramidal cells in the regions indicated by black arrows was decreased and showed an irregular arrangement. The data are expressed as mean ± SD for eight rats per group. * *p* < 0.05, significant difference compared to the control group.

HE-staining and Nissl-staining results showed that, in the control group, the morphology of CA1 and CA3 region in hippocampus was regular and well-organized (Figure 3A). However, in the MeHg exposure group, the layer of pyramidal cells was slightly thinner in the CA1 and CA3 regions of the hippocampus (Figure 3A), and the number of neurons in the CA1 and CA3 regions of the hippocampus were slightly reduced and showed an irregular arrangement (Figure 3B). Histograms of the Nissl body counts in the hippocampus quantified from Nissl staining analysis (Figure 3C). The telomere length in cerebral cortex, hippocampus and hypothalamus were significantly shortened after exposure to MeHg compared with the control (*p* < 0.05; Figure 3D–F).

### 3.4. MeHg-Induced Alterations in the Levels of Serum Melatonin and Urinary aMT6s in Rats

**Figure 4:**
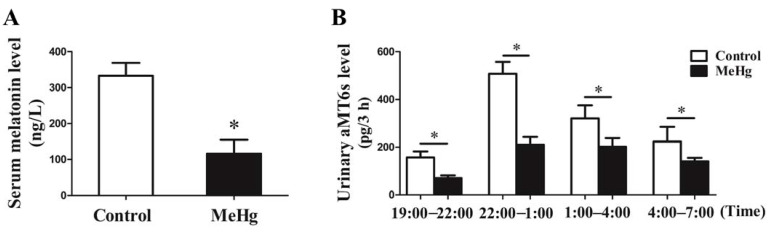
Impact of MeHg exposure on serum melatonin (**A**) and urinary aMT6s (**B**) in rats. MT, melatonin. The data are expressed as mean ± SD for eight rats per group. * *p* < 0.05, significant difference compared to the control group.

The serum level of melatonin in the rats was significantly decreased after MeHg exposure for 3 months (*p* < 0.05; Figure 4A). To further illustrate the impact of MeHg on melatonin metabolism, the aMT6s level in the urine was measured. The results showed that the urinary aMT6s level in rats was also statistically significantly declined in the MeHg-exposure group compared to the control group (*p* < 0.05; Figure 4B).

### 3.5. Influence of MeHg on the Expression of Melatonin Synthetase and 5-HT 

**Figure 5:**
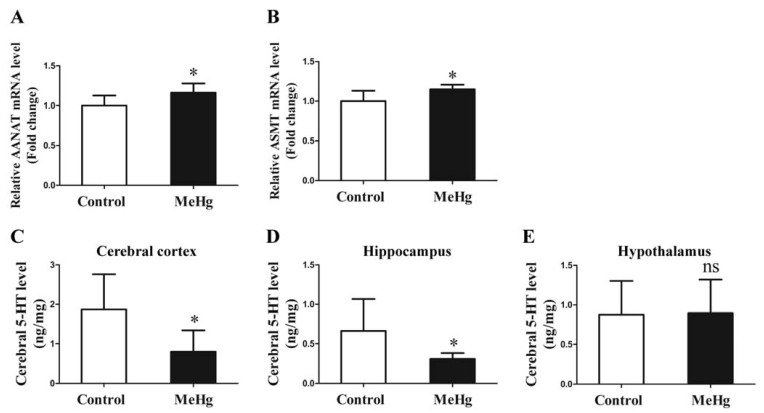
The influence of MeHg exposure on AANAT (**A**) and ASMT (**B**) mRNA expression level and in hippocampal tissue and 5-HT level in cerebral cortex (**C**), hippocampus (**D**) and hypothalamus (**E**). The data are expressed as mean ± SD for eight rats per group. ns, no significance compared to the control group, *p* > 0.05. * *p* < 0.05, significant difference compared to the control group.

To elucidate the cause of declining serum melatonin levels under low-dose MeHg exposure, we further detected the expression of melatonin synthetase in the hippocampus and 5-HT in selected brain regions. The results showed that the mRNA expression levels of melatonin-catalyzing enzymes AANAT and ASMT in the pineal tissue of rats were increased in the MeHg-exposure group compared to the control group (*p* < 0.05; Figure 5A,B). The 5-HT level in the cerebral cortex and hippocampus (*p* < 0.05; Figure 5C,D) was significantly decreased, but there was no obvious difference in the hypothalamus (*p* > 0.05; Figure 5E) after exposure to MeHg when compared with the control.

### 3.6. Correlations of the Level of Serum Melatonin and Urinary aMT6s with Cerebral Telomere Length in Rats

**Figure 6:**
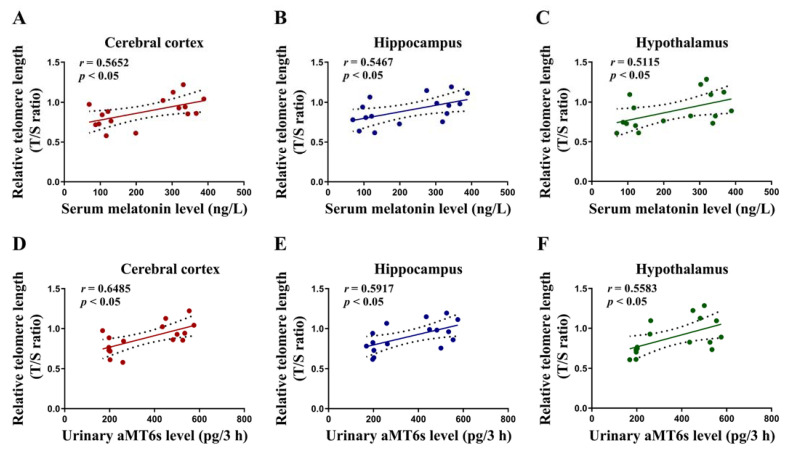
Correlations of serum melatonin and urinary aMT6s level with cerebral telomere length in rats. The serum levels of melatonin were positively correlated with telomere length in cerebral cortex (*r* = 0.5652, *p* < 0.05), hippocampus (*r* = 0.5467, *p* < 0.05) and hypothalamus (*r* = 0.5115, *p* < 0.05) (**A**–**C**), and the urinary levels of aMT6s were positively correlated with telomere length in cerebral cortex (*r* = 0.6485, *p* < 0.05), hippocampus (*r* = 0.5917, *p* < 0.05) and hypothalamus (*r* = 0.5583, *p* < 0.05) (**D**–**F**).

To elucidate the possible causal relationship between cerebral telomere shortening and declined levels of serum melatonin and urinary aMT6s, a correlation analysis was conducted. The results of the correlation analysis showed a positive correlation between serum melatonin level and telomere length in the cerebral cortex, hippocampus and hypothalamus (*p* < 0.05; Figure 6A–C) in the two experiment groups (*n* = 16). There was also a positive correlation between urinary aMT6s level and telomere length in the cerebral cortex, hippocampus and hypothalamus (*p* < 0.05; Figure 6D–F) in the two experiment groups (*n* = 16).

### 3.7. The Hypothesis of Adverse Impacts of MeHg on Cerebral Telomere Length and Causal Relationship with Reduced Melatonin Synthesis and Metabolites 

**Figure 7:**
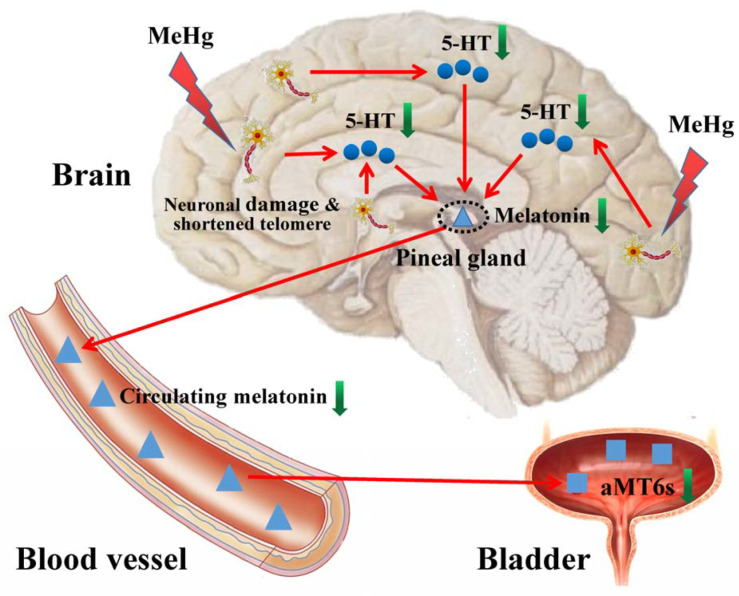
Proposed model of adverse impacts of MeHg on cerebral telomere length and causal relationship with reduced melatonin synthesis and metabolites. The blue dots indicate 5-HT, the blue triangles indicate melatonin and the blue squares indicate aMT6s.

As shown in Figure 7, these results indicate that subchronic low-dose MeHg exposure could cause neuronal damage and telomere shortening, resulting in decreased neuronal synthesis of 5-HT, which, in turn, reduces the level of melatonin synthesis and secretion, as well as the level of aMT6s in urine.

## 4. Discussion

MeHg exposure has been identified as a neurotoxic substance [27] and a risk factor for neurodegenerative disease in humans [7]. Despite knowledge of the adverse effects of high-dose MeHg exposure on the CNS, evidence of the neurotoxic effects of low-dose and continual MeHg exposure is limited. In addition, there is a lack of effective early-warning biomarkers to assess low-dose MeHg-induced CNS damage. 

The neurotoxic effects of MeHg may be significantly related to the exposure dose. Previous studies have indicated that MeHg exposure at high doses (≥1 mg/kg/day) induces neurodevelopmental toxicity and behavioral disorders [28,29]. Valentini and Grotto showed that subchronic MeHg exposure at low doses (0.1 mg/kg/day) decreased the activities of butyrylcholinesterase and antioxidant enzymes in plasma [30,31]. In the present study, subchronic MeHg exposure at dose of 0.1 mg/kg/day decreased the frequency of entering the target quadrant and spending time in the target quadrant in Sprague Dawley rats undergoing a Morris water maze experiment. However, the escape latency showed no statistical significance between the two experimental groups, as shown in Figure 2. Furthermore, low-dose MeHg exposure reduced the number of neurons in hippocampal CA1 and CA3 regions slightly and affected neuronal arrangement in rats. These results suggest that subchronic low-dose MeHg exposure induces a kind of early and indiscoverable neurotoxic effect.

Telomere shortening is a hallmark of cell senescence [32]. However, accelerated telomere shortening and cell senescence in brain cells has been associated with several neurodegenerative diseases, including Parkinson’s and Alzheimer’s diseases [33,34,35]. Investigating the effect of MeHg on the telomere length of brain cells may provide a basis for the prevention of neurological diseases. Although an epidemiological study has shown that MeHg exposure was not associated with leukocyte telomere length in both mothers and their children [36], an animal study revealed that MeHg reduced the expression of telomerase reverse transcriptase (TERT) in mice brains [37]. Our results further suggest that low-dose MeHg exposure for 3 months could significantly reduce the telomere length in the cerebral cortex, hippocampus and hypothalamus in rats. Therefore, the shortened telomere length of brain cells may be an important early manifestation of CNS damage induced by MeHg at low levels.

Melatonin is an important endogenous hormone secreted by the pineal gland [16]. Numerous studies have shown that exogenous melatonin could antagonize mercury-induced neurotoxicity [19,20,21]. To date, however, the effect of MeHg on endogenous melatonin secretion is unknown. In general, the level of serum melatonin was a good indicator, reflecting the secretion of melatonin and, hence, the need to detect the level of serum melatonin. Our results showed that the serum melatonin level in rats exposed to MeHg was significantly lower than those in the control group, indicating that low-dose MeHg may inhibit endogenous melatonin synthesis.

There is a growing body of evidence suggesting that the level of aMT6s in urine could reflect the circulating melatonin [23,24]. In addition, detection biomarkers in urine are non-invasive and more readily available compared to blood; therefore, we further examined the effect of MeHg exposure on the level of melatonin urinary metabolite aMT6s. Previous research has shown that the level of aMT6s in urine reached a peak at 22:00–1:00 a.m. in rats [38,39]. We examined urinary aMT6s levels in four consecutive time periods in rats. In accordance with previous studies, the results showed that the aMT6s level at 22:00–1:00 a.m. was the highest and then declined. Moreover, the urinary aMT6s level in the MeHg-exposure group was lower than that in the control group at the four selected time periods. Therefore, aMT6s level in urine may reflect the circulating melatonin level and melatonin secretion to some extent following MeHg exposure.

To explain the reason for the decline in melatonin secretion induced by MeHg, we focused on two aspects. On the one hand, studies have demonstrated that AANAT and ASMT are two key catalyzing enzymes in the synthesis of melatonin [40] and can catalyze the synthesis of 5-HT into melatonin [41]. Thus, the effect of MeHg on the expression of AANAT and ASMT was detected in the pineal gland. Contrary to expectation, however, the expression of both enzymes increased rather than decreased under MeHg exposure. It is speculated that the increased expression of AANAT and ASMT may be related to the decreased level of melatonin through a type of negative feedback regulation. On the other hand, previous studies have shown that MeHg exposure caused a marked reduction in 5-HT in rat brains [42,43]. The expression of 5-HT in rat brain tissue was further detected. In accordance with previous studies, our results showed that 5-HT level in the cerebral cortex and hippocampus significantly decreased but that there was no obvious difference in the hypothalamus after exposure to MeHg. Since 5-HT was primarily synthesized in neurons and concentrated in synapses in the CNS [44], we hypothesized that the MeHg-induced decline in 5-HT could be attributed to neuronal damage.

Based on the above findings, it was speculated that there may be a correlation between neuron damage and the secretion and metabolism of melatonin under MeHg exposure. Therefore, the correlation between brain tissue telomere length and the level of serum melatonin and aMT6s in urine at peak time (namely, 22:00–1:00 a.m.) was further analyzed. The results showed that the telomere lengths of the three brain regions were all positively correlated with the level of serum melatonin and urinary aMT6s, suggesting that aMT6s in urine may act as an early sensitive indicator of the CNS damage caused by MeHg.

## 5. Conclusions

Taken together, this study reveals that subchronic low-dose MeHg exposure accelerated cerebral telomere shortening in rats. Moreover, low-dose MeHg exposure led to declines in melatonin synthesis and metabolism, possibly due to the reduced 5-HT synthesis as a result of neuronal damage. Importantly, the urinary aMT6s level was positively correlated with a shortened cerebral telomere length, indicating that urinary aMT6s may be an early-warning biomarker to assess low-dose MeHg-induced CNS damage. This study will provide insight into the prevention of CNS damage for populations exposed to MeHg. As the results were from animal experimental research, the association between the degree of CNS damage and urinary aMT6s level in populations exposed to environmental MeHg needs further investigation.

## Data Availability

The data presented in this study are available upon request from the corresponding author.
